# Improvement of Quality of Life by Switching from Oral Semaglutide to Tirzepatide in Patients with Type 2 Diabetes

**DOI:** 10.31662/jmaj.2025-0011

**Published:** 2025-05-26

**Authors:** Takahiro Fukaishi, Fumiaki Ishibashi

**Affiliations:** 1 Department of Diabetes and Metabolism, Koganei Tsurukame Clinic, Tokyo, Japan; 2 Endoscopy Center, Koganei Tsurukame Clinic, Tokyo, Japan; 3 Department of Gastroenterology, International University of Health and Welfare, Ichikawa Hospital, Ichikawa, Japan

**Keywords:** type 2 diabetes, glucagon-like peptide-1 receptor agonist, semaglutide, tirzepatide, DTR-QOL

## Abstract

**Introduction:**

Tirzepatide, a novel dual glucose-dependent insulinotropic polypeptide and glucagon-like peptide-1 receptor agonist has shown significant promise in weight reduction and glycemic management in patients with type 2 diabetes (T2DM). However, there have been no reports evaluating the quality of life (QOL) in patients who switched from oral semaglutide to tirzepatide. This study prospectively investigated short-term changes in the QOL of these patients.

**Methods:**

This study was a single-center, prospective observational study. Participants were patients with T2DM who switched from oral semaglutide to tirzepatide due to insufficient weight reduction between May 1, 2023, and December 31, 2023. The primary outcome was the change in the Diabetes Therapy-Related QOL (DTR-QOL) questionnaire scores from baseline to 3 months after switching. The changes in glycemic management and body weight were also assessed as secondary outcomes.

**Results:**

Eleven patients were enrolled in this study. The total DTR-QOL score was significantly improved 3 months after switching (from 58.0 ± 9.9 to 81.5 ± 11.2, p < 0.001). Four subscale domains were also significantly improved (p < 0.001, p < 0.001, p = 0.015 and p < 0.001, respectively). There were no significant changes in glycated hemoglobin levels and body weight 3 months after switching (from 6.8 ± 1.0% to 6.4 ± 0.8%, p = 0.068 and from 86.5 ± 19.6 kg to 84.2 ± 19.7 kg, p = 0.099, respectively).

**Conclusions:**

Switching from oral semaglutide to tirzepatide potentially improves overall QOL in patients with T2DM in a short period of time. Further large cohort studies will reinforce the results.

## Introduction

Obesity is a significant risk factor for the development and progression of type 2 diabetes (T2DM). The prevalence of obesity has been rising globally, contributing to an increase in T2DM cases ^(1)^. Obesity exacerbates insulin resistance, leading to higher blood glucose levels and increased cardiovascular risk ^(2)^. Effective management of T2DM in patients with obesity requires a multifaceted approach that addresses both hyperglycemia and excess weight.

Tirzepatide, a novel dual glucose-dependent insulinotropic polypeptide and glucagon-like peptide-1 (GLP-1) receptor agonist weekly administered subcutaneously, has shown significant promise in the management of T2DM with efficient weight reduction ^(3)^. The efficacy of tirzepatide has been extensively evaluated in the SURPASS clinical trial program, which includes a series of randomized controlled trials comparing tirzepatide with various standard treatments for T2DM. The SURPASS-2 trial, for instance, demonstrated that tirzepatide achieved superior glycemic management and greater weight reduction compared to subcutaneous semaglutide, another GLP-1 receptor agonist, in patients with T2DM inadequately managed with metformin alone ^(4), (5)^. Additionally, the SURPASS-3 trial highlighted the benefits of tirzepatide in improving glycated hemoglobin levels and promoting significant weight loss in patients with T2DM, compared to insulin degludec ^(6)^. These findings suggest that tirzepatide could offer a more effective therapeutic option for patients struggling with both glycemic management and obesity, potentially leading to improved overall metabolic outcomes. From the results of the SURPASS-2 trial, we can speculate that tirzepatide could be an effective novel treatment option for patients with T2DM who could not achieve sufficient glycemic management or weight reduction with oral semaglutide.

Quality of life (QOL) for patients with T2DM is profoundly influenced by the treatment method used. The Diabetes Therapy-Related QOL (DTR-QOL) questionnaire is a validated tool that assesses treatment satisfaction and its impact on the patient QOL ^(7), (8)^. Studies have shown that newer treatment modalities, such as GLP-1 receptor agonists, not only improve glycemic management but also enhance patient-reported outcomes. For instance, it was reported that newly started liraglutide significantly improved glycemic control and reduced body weight without deteriorating QOL in obese patients with T2DM ^(9)^. Other studies have shown that the switching from basal-bolus insulin therapy to a combination of dulaglutide and basal insulin, and the switching from daily dipeptidyl peptidase-4 (DPP-4) inhibitors to weekly trelagliptin in patients with T2DM improved the DTR-QOL scores ^(10), (11)^. Furthermore, the introduction of tirzepatide has demonstrated an improvement in the scores of the DTR-QOL compared with dulaglutide ^(12)^. These findings highlight that optimizing treatment regimens based on patient preferences and QOL can lead to better adherence and overall improved outcomes. Switching from oral semaglutide to tirzepatide can be a promising treatment option if patients are adherent to the administration of tirzepatide. However, it is expected to cause changes in patient QOL due to the initiation of an injectable medication and its effect on appetite or gastrointestinal movement, which might result in poorer metabolic outcomes than expected through decreased patient adherence. However, there have been no real-world reports evaluating QOL in patients who switched from oral semaglutide to tirzepatide. In this study, we prospectively evaluated the changes in patient QOL after switching oral semaglutide to tirzepatide using the DTR-QOL questionnaires.

## Materials and Methods

### Study design

This study was a single-center, prospective observational study. Written informed consent was obtained from each patient before enrollment in the study. This study was carried out following the Declaration of Helsinki.

### Participants

Participants were Japanese patients with T2DM who switched from oral semaglutide to tirzepatide due to insufficient weight reduction between May 1, 2023, and December 31, 2023, at Koganei Tsurukame Clinic. Patients using oral semaglutide who have not achieved a 5% weight reduction in the past 6 months are defined as having insufficient weight reduction. All participants were adherent to oral semaglutide in terms of both adverse effects and administration methods. We excluded patients with type 1 diabetes, acute or chronic pancreatitis, the experience of injection therapy for diabetes, body mass index (BMI) lower than 23 kg/m^2^, and hypersensitivity to the drugs used in this study.

### Outcomes

The primary outcome of this study was the change in the DTR-QOL scores from baseline to 3 months after switching. The changes in glycemic management and body weight 3 months after switching were the secondary outcomes.

### Procedures

Tirzepatide was started at 2.5 mg, increased to 5 mg after 4 weeks, and then maintained at the same dose due to the supply restrictions of tirzepatide in Japan during the study period. Diet and exercise were maintained as usual and no changes were made to the other diabetes medications in any of the cases during the study period. Nurses provided instruction on injection techniques at the initiation of tirzepatide and occasionally confirmed the technique during subsequent visits. We monitored adherence to tirzepatide by collecting the used injection pens from patients at our clinic during their visits. Adverse effects, such as hypoglycemia and gastrointestinal symptoms, were monitored during the trial. Hypoglycemia was defined as blood glucose <70 mg/dL or the presence of hypoglycemic symptoms.

After written informed consent was obtained, the DTR-QOL was performed at the switching (baseline) and 3 months after switching. The patients filled out the questionnaires by themselves in private to avoid any influence from physicians or medical care providers. The changes in the scores on both questionnaires from baseline to 3 months after switching were compared between the two groups.

### DTR-QOL

The DTR-QOL was established to evaluate patient QOL ^(7), (8)^. The DTR-QOL includes 29 items, and responses are scored on a 7-point scale, from +7 to +1. The assessment covers four domains: domain 1, burden on social activities and daily activities; domain 2, anxiety and dissatisfaction with treatment; domain 3, hypoglycemia; and domain 4, satisfaction with treatment. Total DTR-QOL score and subscale scores were calculated as shown below and were converted to a score of a maximum of 100 (highest QOL). Total DTR-QOL score = (overall points from questions 1 to 29) × (100/203). Subscale scores were calculated in the same way: domain 1 = (total points from questions 1 to 13) × (100/91); domain 2 = (total points from questions 14 and 19 to 25) × (100/56); domain 3 = (total points from questions 15 to 18) × (100/28); and domain 4 = (total reversed points from questions 26 to 29) × (100/28).

### Statistical analyses

Results are expressed as means ± standard deviation. Each domain of DTR-QOL scores, glycated hemoglobin levels, body weight, and BMI were tested for normality using the Shapillo-Wilk test. Differences between the values before the initiation of tirzepatide and values after that were evaluated using the paired t-test, if these followed a normal distribution, or the Wilcoxon signed-rank test, if not. p Values <0.05 were considered statistically significant. Data were analyzed using Microsoft Excel 2016 for Windows and EZR Version 1.68 for Windows.

## Results

### Patient characteristics

Eleven patients (two men and nine women), who switched oral semaglutide to tirzepatide due to insufficient weight reduction, were enrolled in the study, and all patients adhered to the treatment schedule and completed the study. The baseline clinical and metabolic characteristics are shown in Table 1. The mean age of the participants was 50.1 ± 8.6 years, with the mean glycated hemoglobin of 6.8 ± 1.0%, the mean weight of 86.5 ± 19.6 kg, and the mean BMI was 32.4 ± 4.9 kg/m^2^. Nine patients switched from oral semaglutide 14 mg to tirzepatide, and two patients switched from oral semaglutide 7 mg to tirzepatide.

**Table 1.** Patient Characteristics at Baseline.

N	11
Age (years)	50.1 ± 8.6
Sex (male/female)	2/9
Body weight (kg)	86.5 ± 19.6
Body mass index (kg/m^2^)	32.4 ± 4.9
Glycated hemoglobin (%)	6.8 ± 1.0
Diabetes medications other than tirzepatide
Biguanide (n)	9
SGLT2 inhibitor (n)	6
Sulfonylurea (n)	1
Thiazolidinedione (n)	1
Alpha-glucosidase inhibitor (n)	1
None (n)	2

SGLT2: sodium-glucose cotransporter 2

### Changes in the DTR-QOL scores

**Figure 1.**
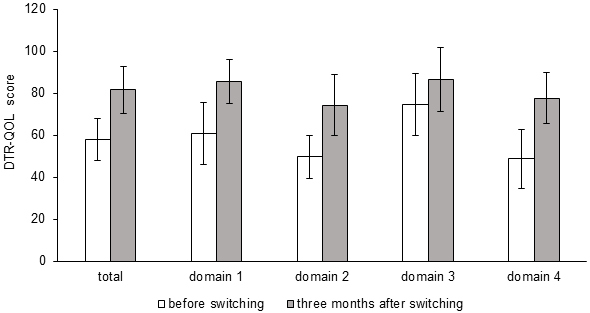
Changes in total/subscale Diabetes Therapy Related Quality of Life (DTR-QOL) scores from baseline to 3 months after switching. The subscale assessment covers the following four domains: domain 1, burden on social activities and daily activities; domain 2, anxiety and dissatisfaction with treatment; domain 3, hypoglycemia; and domain 4, satisfaction with treatment.

The DTR-QOL scores, the primary outcome of this study, were assessed in all 11 patients, and these were significantly improved 3 months after switching in total score (from 58.0 ± 9.9 to 81.5 ± 11.2, p < 0.001) and all four subscale domains: domain 1, burden on social activities and daily activities; domain 2, anxiety and dissatisfaction with treatment; domain 3, hypoglycemia; and domain 4, satisfaction with treatment (from 60.8 ± 14.8 to 85.6 ± 10.7, p < 0.001, from 49.7 ± 10.4 to 74.4 ± 14.4, p < 0.001, from 74.7 ± 14.9 to 86.7 ± 15.1, p = 0.015 and from 48.7 ± 14.1 to 77.6 ± 12.1, p < 0.001, respectively) (Figure 1). The changes in the scores of each questionnaire are shown in Table 2.

**Table 2.** Results of Each DTR-QOL Questionnaire.

Domain	Questions		Score		Difference	*p* value
			Baseline	3 months		
Domain 1:burden on social activities and daily activities	Q1	My current diabetes treatment interferes with my work and activities	4.2±1.7	5.2±1.4	+1.0	0.008
	Q2	My current diabetes treatment limits the scope of my activities	4.3±1.4	6.3±1.1	+2.0	<0.001
	Q3	It is difficult to find places on time for my current diabetes treatment	4.9±1.4	6.5±1.9	+1.6	0.019
	Q4	My current diabetes treatment interferes with group activities and personal friendships	4.5±1.2	6.6±0.6	+2.1	<0.001
	Q5	It is a burden getting up at a certain time every morning for my current diabetes treatment	4.0±2.0	6.4±1.0	+2.4	0.003
	Q6	With my current diabetes treatment, the restricted meal times are a burden	3.5±1.8	6.4±0.8	+2.9	<0.001
	Q7	When I eat out, it is difficult to manage my current diabetes treatment	4.6±2.0	6.0±1.2	+1.4	0.023
	Q8	I feel like my current diabetes treatment takes away the enjoyment of eating	4.5±1.6	5.8±1.1	+1.3	0.024
	Q9	With my current diabetes treatment, it is hard to curb my appetite	3.2±1.3	4.4±2.0	+1.2	0.071
	Q10	The time and effort to manage my current diabetes treatment are a burden	3.7±1.4	6.2±1.1	+2.5	0.002
	Q11	I am constantly concerned about time to manage my current diabetes treatment	4.5±1.4	6.2±0.9	+1.7	0.048
	Q12	Pain due to my current diabetes treatment is uncomfortable	5.1±1.7	6.0±1.0	+0.9	0.203
	Q13	Gastrointestinal symptoms (nausea, passing gas, diarrhea, abdominal pain) due to my current diabetes treatment are uncomfortable	4.3±1.8	6.0±1.6	+1.7	0.047
Total score of domain 1			55.4±13.5	77.9±9.7	+22.5	<0.001
Domain 2: anxiety and dissatisfaction with treatment	Q14	I am bothered by weight gain with my current diabetes treatment	3.5±1.2	5.9±1.2	+2.4	0.004
	Q19	I have uncomfortable symptoms due to hyperglycemia (high blood glucose)	5.0±1.5	6.2±1.1	+1.2	0.034
	Q20	I am worried about high blood glucose	3.0±1.3	4.9±1.8	+1.9	0.006
	Q21	I am dissatisfied that my blood glucose is unstable (high and low)	3.8±1.4	5.5±1.2	+1.7	0.013
	Q22	I am worried that complications might get worse with my current diabetes treatment	3.5±1.7	4.9±1.8	+1.4	0.027
	Q23	I get anxious thinking about living while on my current diabetes treatment	3.3±1.7	4.6±1.4	+1.3	0.035
	Q24	I find it unbearable to think that even if I continue my current diabetes treatment, my diabetes may not be cured	3.0±1.5	5.0±1.4	+2.0	0.002
	Q25	I am concerned that if I continue my current diabetes treatment, the efficacy may diminish	3.1±1.2	4.5±1.7	+1.4	0.009
Total score of domain 2			27.8±5.8	41.6±8.1	+13.8	<0.001
Domain 3: hypoglycemia	Q15	I worry about low blood glucose due to my current diabetes treatment	4.7±1.5	5.8±1.3	+1.1	0.040
	Q16	I am scared because of low blood glucose	5.3±1.2	6.4±1.0	+1.1	0.024
	Q17	I am sometimes bothered by low blood glucose	5.5±1.2	6.1±1.2	+0.6	0.216
	Q18	Symptoms due to low blood glucose are uncomfortable	5.4±1.1	6.0±1.3	+0.6	0.409
Total score of domain 3			20.9±4.2	24.3±4.2	+3.4	0.015
Domain 4: satisfaction with treatment	Q26	Overall, I am satisfied with my current blood glucose management	3.2±0.8	5.6±1.1	+2.4	<0.001
	Q27	With my current diabetes treatment, I am confident that I can maintain good blood glucose management	3.1±0.9	5.0±1.0	+1.9	0.005
	Q28	I am hopeful about the future with my current diabetes treatment	3.8±1.3	5.5±1.0	+2.3	0.013
	Q29	With regards to diabetes treatment, I am satisfied with current treatment methods	3.5±1.4	5.6±0.9	+2.1	0.001
Total score of domain 4			13.6±4.0	21.7±3.4	+8.1	<0.001
Total score of all items			117.7±20.2	165.5±22.8	+47.8	<0.001

Scores are shown as the mean ±SD *p* values are the results of Wilcoxon signed-rank test

### Metabolic parameters

**Figure 2.**
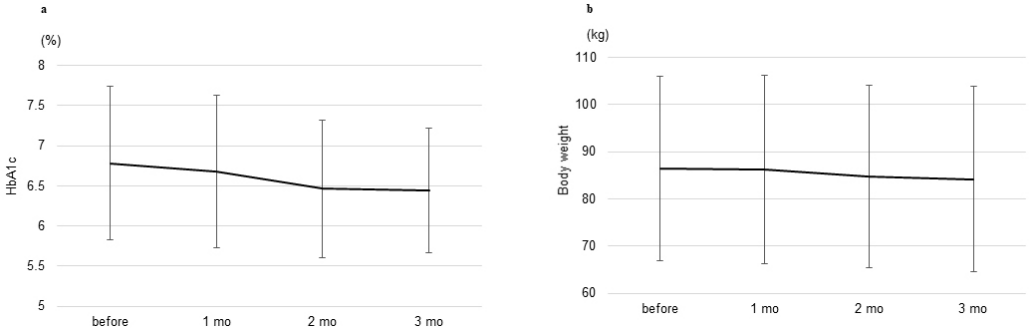
a: Changes in glycated hemoglobin levels during the study period. b: Changes in body weight during the study period. mo: month(s).

The changes in glycemic management and body weight 3 months after switching, the secondary outcome of this study, were assessed. As shown in Figure 2a, there were no significant changes in glycated hemoglobin levels from baseline to 3 months after switching (from 6.8 ± 1.0% to 6.4 ± 0.8%, p = 0.068). There was no significant difference in the mean change in body weight and BMI from baseline 3 months after switching (from 86.5 ± 19.6 kg to 84.2 ± 19.7 kg, p = 0.099 and from 32.4 ± 4.9 kg/m^2^ to 31.6 ± 5.5 kg/m^2^, p = 0.101, respectively). (Figure 2b).

### Adverse effects

No hypoglycemic episodes were observed during the study period. None of the other major adverse effects, such as local reaction, drug eruption, and severe gastrointestinal symptoms, were also observed during the study period. As minor adverse effects, mild diarrhea occurred after the initiation of tirzepatide in three patients but gradually improved. In addition, mild nausea occurred after the initiation of tirzepatide in two patients, but they reported that it was milder than what they had experienced with oral semaglutide.

## Discussion

For efficient diabetes treatment, it has been shown that optimizing a treatment regimen that takes into consideration the patient’s QOL is crucial for improving patient adherence, and consequently, for improving overall metabolic outcomes. From the results of the SURPASS-2 trial, we can speculate switching from oral semaglutide to tirzepatide can be a promising treatment option in terms of glycemic management and weight reduction. In our clinic, among the patients who were prescribed oral semaglutide in 2024, 15.8% underwent switching to tirzepatide. This also suggests that switching from oral semaglutide to tirzepatide has recently become a common clinical practice. As sustained weight reduction has been shown to be linked to the maintenance of diabetes remission ^(13)^, achieving efficient weight reduction with tirzepatide while enhancing QOL is considered to be crucially beneficial in the treatment of T2DM. However, switching from oral semaglutide to tirzepatide can affect patient QOL which might result in poorer metabolic outcomes than expected through decreased patient adherence. There have been no real-world reports evaluating QOL in patients who switched from oral semaglutide to tirzepatide. In this study, we first demonstrated that switching from oral semaglutide to tirzepatide improved patient QOL without significant improvement of glycated hemoglobin levels and weight reduction.

It has been reported that tirzepatide significantly improves patient QOL, especially when used in higher doses or when there are substantial improvements in glycemic management and weight reduction ^(14), (15)^. Other studies have also reported that initiating liraglutide in T2DM patients who have never used injectable medications improves patient QOL, despite the initiation of injection and gastrointestinal adverse effects, through significantly improved glycemic management and weight reduction ^(9)^. However, this study demonstrated that even when the dose of tirzepatide is increased only up to 5 mg and without significant improvements in glycemic management and weight reduction, patient QOL still significantly improved compared to oral semaglutide.

Oral semaglutide should be taken on an empty stomach upon waking with a sip (≤ 120 mL) of plain water and at least 30 minutes before the first food, beverage, or other oral medications of the day for efficient absorption in the stomach. Many patients adapt to this method, but occasionally we experience cases who feel burdened by it. By switching from oral semaglutide to tirzepatide, patients can stop this daily routine, which might contribute to the improvement of domain 1 (burden on social activities and daily activities) in the DTR-QOL. In contrast, there was no significant difference in Q9 (With my current diabetes treatment, it is hard to curb my appetite) and Q12 (Pain due to my current diabetes treatment is uncomfortable) in domain 1. Considering this point, the improvement in patient QOL observed after switching from oral semaglutide to tirzepatide is likely largely attributed to the reduction of the burden associated with taking oral semaglutide. In the previous report, switching from daily DPP-4 inhibitors to a weekly DPP-4 inhibitor could partially improve treatment satisfaction levels in patients with T2DM without affecting glycemic control and body weight ^(11)^. By reducing the burden of treatment through switching from daily medication to weekly medication, it might be possible to improve patient QOL, even if significant improvements in glycemic management or weight loss are not achieved. In addition, there was a significant improvement in Q13 (Gastrointestinal symptoms [nausea, passing gas, diarrhea, abdominal pain] due to my current diabetes treatment are uncomfortable) in domain 1, indicating that the switch from oral semaglutide to tirzepatide contributed to the improvement in patient QOL from the perspective of adverse effects. In the SURPASS-2 trial, there was no significant difference in gastrointestinal-related adverse events, such as nausea and diarrhea, between tirzepatide and semaglutide ^(4)^. However, in this study, several patients reported experiencing less nausea with tirzepatide compared to oral semaglutide. Uncovering the detailed mechanisms of tirzepatide and semaglutide in appetite suppression will further clarify this aspect.

In domain 2 (anxiety and dissatisfaction with treatment) and 4 (satisfaction with treatment) in the DTR-QOL, there were significant improvements in all questionnaires. This result might reflect a trend of improvement in glycemic management and weight reduction by switching from oral semaglutide to tirzepatide, although not significant. This suggests that even non-significant improvements in glycemic management and weight reduction might contribute to encouraging and motivating diabetes treatment, consequently leading to an improvement in patient QOL. In addition, nurses in our clinic provided instruction on injection techniques at the initiation of tirzepatide and occasionally confirmed the technique during subsequent visits. All patients demonstrated good mastery of the injection technique, and it is presumed that the careful guidance from nurses contributed to an improved QOL by the improvement in satisfaction with treatment. However, no patients received dietary therapy follow-up from a dietitian during the study period. We assume that providing nutritional guidance after achieving appetite suppression with tirzepatide could make dietary therapy more effective and contribute to improving QOL.

In this study, no hypoglycemic episodes were observed during the study period including one patient using sulfonylurea. Unexpectedly, switching from oral semaglutide to tirzepatide resulted in the improvement of domain 3 (hypoglycemia) in the DTR-QOL as in other domains. We consider that the increase in domain 3 score reflected the overall improvement in clinical parameters and QOL without a rise in hypoglycemic events.

The questionnaire regarding pain (Q12), which would typically be expected to worsen with the initiation of injectable medication, actually showed a trend toward improvement with the initiation of tirzepatide. This may be because the pain from a once-weekly subcutaneous injection is tolerable for many patients.

In this study, we prospectively demonstrated that patient QOL significantly improved as early as 3 months after switching from oral semaglutide to tirzepatide. Early QOL improvement following a treatment change might contribute to the motivation of continuation of long-term treatment through enhanced patient adherence. As diabetes treatment can be a long-term process in a patient’s whole life, it is important to choose treatments that can keep better patient QOL in the long term.

This study has several limitations. First, it is a single-center study with a small sample size. In addition, the dose of tirzepatide could only be increased up to 5 mg due to the supply restrictions of tirzepatide in Japan, which may have resulted in insufficient glycemic management and weight reduction, potentially affecting the accurate assessment of patient QOL. Therefore, the generalizability of this study’s findings might be limited. A larger cohort study will help verify the validity of these findings. Second, there was a gender imbalance, with 80% of the participants being female, which may have led to an insufficient assessment of QOL in males. A previous study investigating psychological resistance to insulin therapy in patients with T2DM revealed that women exhibited significantly stronger psychological resistance than men ^(16)^. The study suggested that this could be due to women having a greater fear of injections and being more concerned about how they are perceived by others when administering injections. Considering this point, the significant improvement in patient QOL observed after switching from oral medication to injectable medication in a population with a high proportion of women is noteworthy. However, since many factors besides pain can affect patient QOL, a more balanced sampling with fewer gender differences would have been preferable to minimize potential biases. Third, we evaluated patient QOL over a short period of 3 months, but we have not assessed QOL over a longer period. In this study, there were no significant improvements in glycemic management or weight reduction 3 months after switching from oral semaglutide to tirzepatide. However, longer-term use or higher doses of tirzepatide can improve glycemic management and weight reduction, which could also lead to better patient QOL. The short duration of this study may underestimate the longer-term QOL or metabolic benefits of tirzepatide. Studies with a longer follow-up period are necessary for more accurate interpretation.

In conclusion, we demonstrated that switching from oral semaglutide to tirzepatide improved overall patient QOL in a short period. The finding of this study that patient QOL improved despite the switching from oral medication to injectable medication provides an insight that makes the initiation of tirzepatide a more practical option for clinicians.

## Article Information

### Conflicts of Interest

None

### Author Contributions

Takahiro Fukaishi conceived and designed the study. Takahiro Fukaishi wrote the original draft. Fumiaki Ishibashi interpreted the data and revised the manuscript for valuable intellectual content. Takahiro Fukaishi and Fumiaki Ishibashi agree with the content of the manuscript. Fumiaki Ishibashi is the guarantor of this work, has full access to all the data in the study, and takes responsibility for the integrity of the data and the accuracy of the data analysis.

### Approval by Institutional Review Board (IRB)

The protocol of this study was collectively reviewed and approved by the ethical review committee of Tsurukame-kai (approval number: 2305, approved on 23 May 2023).
